# Women’s perceptions of PERSPECTIVE: a breast cancer risk stratification e-platform

**DOI:** 10.1186/s13053-022-00214-4

**Published:** 2022-02-24

**Authors:** Saima Ahmed, Emmanuelle Lévesque, Rosalind Garland, Bartha Knoppers, Michel Dorval, Jacques Simard, Carmen G. Loiselle

**Affiliations:** 1 Division of Experimental Medicine, McGill University, Montréal, QC Canada; 2 McGill University Centre of Genomics and Policy, Montréal, QC Canada; 3 Medical Surgical Intensive Care Unit, Jewish General Hospital, Montreal, QC Canada; 4 Université Laval, Québec City, QC Canada; 5 CHU de Québec-Université Laval Research Centre, Québec City, QC Canada; 6 CIUSSS Centre-Ouest Montréal, Segal Cancer Centre, Jewish General Hospital, Montreal, QC Canada; 7 Department of Oncology and Ingram School of Nursing, Faculty of Medicine and Health Sciences, McGill University, 680 Sherbrooke Ouest, Office 1812, Montréal, QC H3A 2M7 Canada

**Keywords:** Breast cancer, cancer risk stratification, cancer prevention and screening, e-health

## Abstract

**Background:**

Breast cancer risk stratification categorizes a woman’s potential risk of developing the disease as near-population, intermediate, or high. In accordance, screening and follow up for breast cancer can readily be tailored following risk assessment. Recent efforts have focussed on developing more accessible means to convey this information to women. This study sought to document the relevance of an informational e-platform developed for these purposes.

**Objective:**

To begin to assess a newly developed breast cancer risk stratification and decision support e-platform called PERSPECTIVE (PErsonalised Risk Stratification for Prevention and Early deteCTIon of breast cancer) among women who do not know their personal breast cancer risk (Phase 1). Changes (pre- and post- e-platform exposure) in knowledge of breast cancer risk and interest in undergoing genetic testing were assessed in addition to perceptions of platform usability and acceptability.

**Methods:**

Using a pre-post design, women (*N* = 156) of differing literacy and education levels, aged 30 to 60, with no previous breast cancer diagnosis were recruited from the general population and completed self-report e-questionnaires.

**Results:**

Mean e-platform viewing time was 18.67 min (SD 0.65) with the most frequently visited pages being breast cancer-related risk factors and risk assessment. Post-exposure, participants reported  significantly higher breast cancer-related knowledge (*p* < .001). Increases in knowledge relating to obesity, alcohol, breast density, menstruation, and the risk estimation process remained even when sociodemographic variables age and education were controlled. There were no significant changes in genetic testing interest post-exposure. Mean ratings for e-platform acceptability and usability were high: 26.19 out of 30 (SD 0.157) and 42.85 out of 50 (SD 0.267), respectively**.**

**Conclusions:**

An informative breast cancer risk stratification e-platform targeting healthy women in the general population can significantly increase knowledge as well as support decisions around breast cancer risk and assessment. Currently underway, Phase 2, called PERSPECTIVE, is seeking further content integration and broader implementation .

## Introduction

All women are at risk of developing breast cancer [1]. In both incidence and mortality rates, breast cancer remains the most common cancer among women worldwide [2]. An increased understanding of the underlying factors that contribute to the development of breast cancer has given rise  to the development of public health strategies for prevention and early detection [3, 4]. Given that most women overestimate their risk [5], providing information tailored to specific risk levels may increase adherence to personalized prevention and screening recommendations through informed decision-making [6].

Breast cancer risk stratification follows a disease screening and prevention approach whereby women from a targeted population are classified into subgroups based on their risk of developing the illness [7]. Using the Breast and Ovarian Analysis of Disease Incidence and Carrier Estimation Algorithm (BOADICEA), the risk specific to each woman is assessed based on a combination of factors - including rare mutations in breast cancer predisposition genes, polygenic risk score determined using common low penetrance genetic variants, familial history of related cancers, reproductive history, alcohol, body mass index, hormones, and breast density, then risk is classified into either 1) near population, 2) intermediate, or 3) high [8]. Based on the specific risk level, appropriate screening and prevention measures are recommended [9, 10].

To complement this process, breast cancer information, tailored education, and support are essential in raising awareness and following up on recommendations [11]. Of note, the integration of e-platforms presents the opportunity to provide relevant evidence-based information in a more accessible, sustainable, and efficient manner [12, 13]. For these to work as intended, they must be high quality, relevant, and user-friendly. Moreover, personal health literacy, defined as an individual’s ability to “find, understand, and use information and services to inform health-related decisions and actions for themselves and others”, emphasizes the importance of using health information rather than merely understanding it [14]. Lower health literacy is associated with  reduced use of preventive services [15], especially mammography and screening [16, 17]. Thus, it is essential for breast cancer prevention programs to be accessible to women across different literacy levels [17].

In a randomized controlled trial published in The Lancet, Hersch et al. [18] reported on the use of decision aids for women regarding breast cancer screening. Womenfrom the general population were randomized to receive either 1) The intervention: A decision-aid on screening also may contain information on potential harms such as over detection and false positives, in addition to positive information pertaining to the reduction of breast cancer mortality reduction, or 2) The control condition: A routinely used screening decision aid without reference to potential harms. Results showed significant differences between groups with significantly more women in the intervention group meeting the threshold for enhanced knowledge and informed choice regarding screening post intervention [18]. These changes remained at the 2-year follow up [19].

In addition, the literature supports that decision-making, such as the choice to undergo genetic testing for breast cancer, can be influenced by prior knowledge and emotions [20]. Emotions, for instance, provide quick incentives in , determining importance and value that aid in decision-making, when time, motivation and/or information is limited [20]. Moreover, as the affect-as-information model states – an individual may consider feelings and/or mood as baseline information that ultimately influence how other types of information are processed [21–23]. Thus, underlying emotions can influence a person’s experience interacting with health information, and is assessed herein using positive and negative affect (mood).

### PERSPECTIVE

PERSPECTIVE (PErsonalized Risk Stratification for Prevention and Early deteCTIon of breast cancer) is a Quebec-based project that aims to promote early detection of breast cancer in a cost-effective manner through risk stratification and communication e-tools. It broadens the reach of mammography screening programs to target women, especially those under 50, who may be at higher risk of developing breast cancer but are overlooked due to higher age-based screening guidelines.

**Fig. 1:**
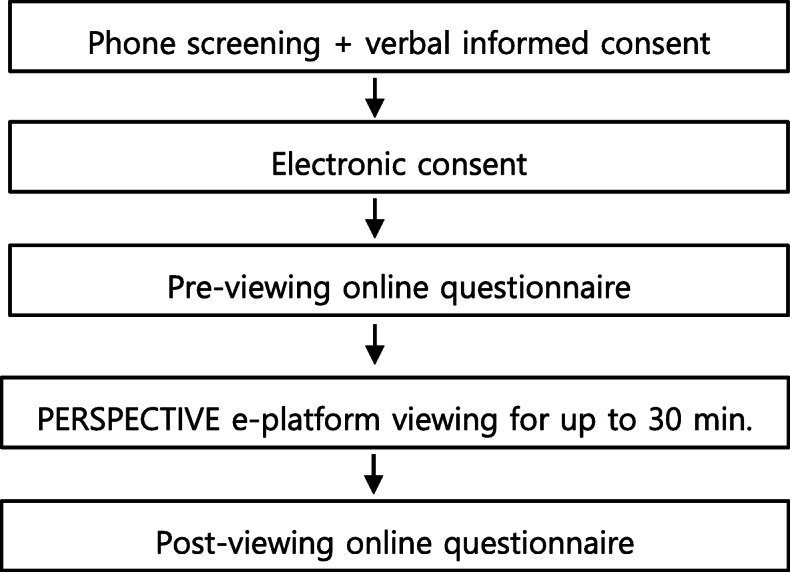
Overview of the study procedures

In Phase 1, a web-based e-platform was developed. This initiative sought to provide comprehensive information on breast cancer risk in an optimal manner to women of different literacy and education levels (Fig. 1). PERSPECTIVE can be used as a pre-assessment means to support women in the decision to have their personal breast cancer risk assessed and post-assessment tool providing personalized recommendations based on risk level once determined.

The password protected e-platform, available in English and French, contains 28 pages of content and images on breast cancer risk stratification, risk factors, prevention, personal risk assessment, risk classification levels, and recommended screening measures. All content was developed in accordance with the *Clinical Advisory Committee on Breast Cancer Screening and Prevention* [24], and revised through an earlier pilot phase of the study which examined a preliminary version of the platform according to feedback from healthy women (*N* = 10) through in-depth semi-structured interviews. These women reported that the e-platform was relevant, easy to navigate, and that they learned new information on their relative breast cancer risk. Presented herein, Phase 1 utilized PERSPECTIVE as a pre-assessment tool to be used by women with no knowledge of their personal breast cancer risk. Thus, family history was not collected and personal risk assessment was not calculated.

### Aims and objectives

The aim of the current study was to begin to assess the PERSPECTIVE e-platform among women in the general population of various literacy and education levels, who were not aware of their personal breast cancer risk.

***Objective 1***: To assess participants changes (pre- and post- e-platform exposure) in knowledge of breast cancer risk, while controlling for sociodemographic variables of age and education.

***Objective 2:*** To assess participants changes (pre- and post- e-platform exposure) in understanding and interest in subsequent genetic testing.

***Objective 3:*** To determine the usability and acceptability of the e-platform. The former refers to the quality of a person’s experience interacting with content, including ease of learning, efficiency of use, memorability, error frequency and severity, and subjective satisfaction [25]. Acceptability refers to how well the e-platform is received by its target audience and the extent to which platformcomponents meet users' needs [26].

## Methods

### Design

A pre-post design was used whereby measures were collected before and after participants interacted with the e-platform.

### Participants, setting, and procedures

Individuals were eligible to participate if they were female, aged between 30 and 60, with no previous breast cancer diagnosis, and had unrestricted access to the internet. Eligibility regarding age was determined in accordance with screening recommendations using a consensus-based method for the risk-classification approach of PERSPECTIVE, whereby women at high risk should begin screening at age 30 [24]. All participants were recruited in Montreal and Quebec City within the province of Quebec, Canada through: (1) flyers and booths in high traffic areas such as grocery stores, malls, school campuses, athletic, and health centers, (2) media such as online newspaper ads, and social media posts on the platforms of community organizations, (3) the research team website (loisellelab.ca). The institutional review boards of the CHU de Québec - Université Laval and Centre intégré universitaire de santé et de services social (CIUSSS) du Centre-Ouest-de-I’Ile-de-Montreal approved the study.

**Fig. 2:**
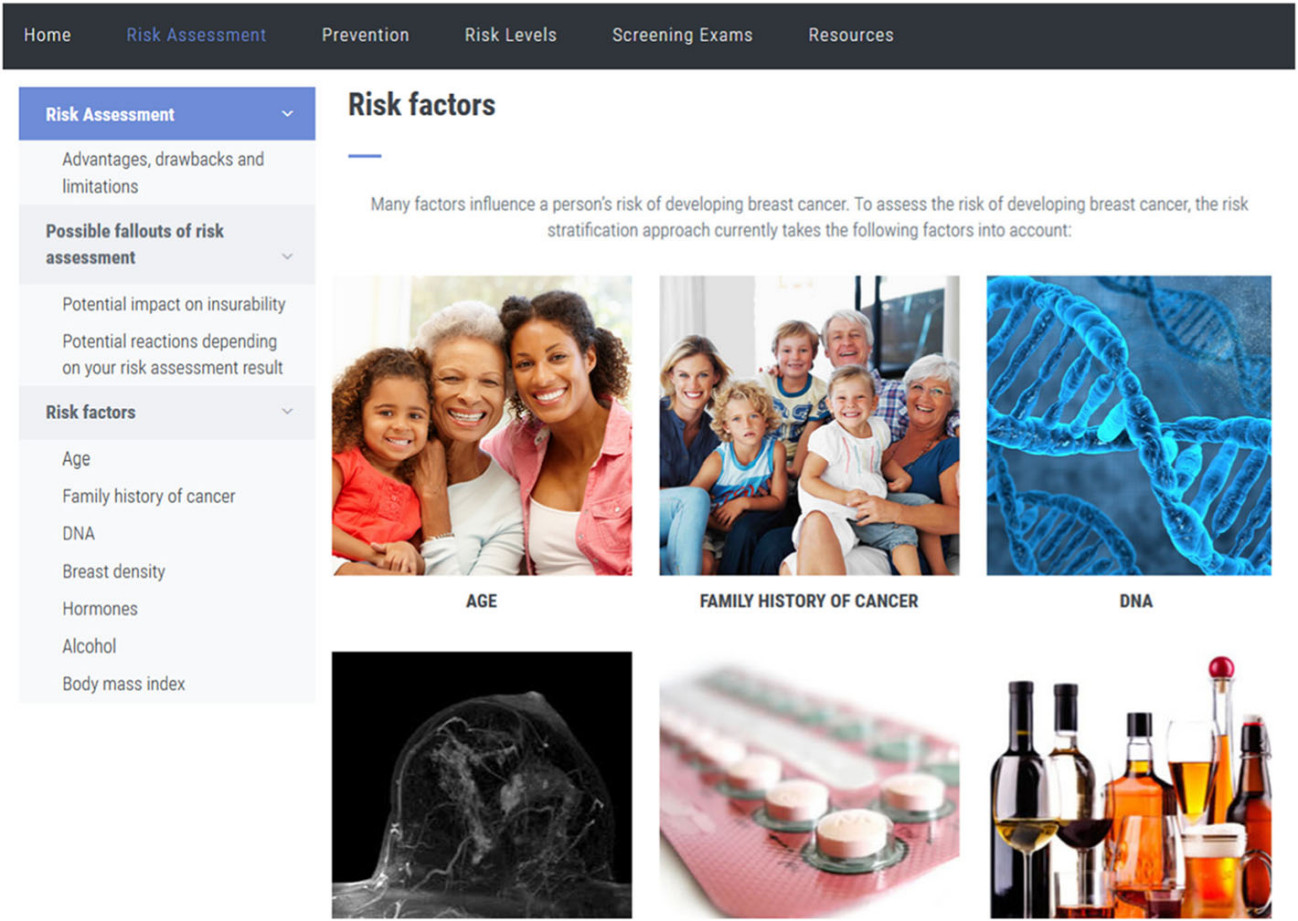
PERSPECTIVE e-platform

Individuals interested in learning more about the study contacted the study team through email or telephone. A team member answered questions and screened for eligibility. At the time of eligibility, participants were made aware that the e-platform was available to be viewed in English and/or French, the choice of language being theirs. If eligible, informed consent was obtained over the phone, then electronically. Following consent, a username and password for the PERSPECTIVE e-platform were provided. Participants completed the pre-exposure online questionnaire on LimeSurvey before viewing the e-platform for up to 30 min. The post-exposure questionnaire was completed immediately after e-platform viewing. Figure 2 provides an overview of study procedures. Participants had the choice of completing questionnaires in French or English.

### Measures

#### Breast cancer risk knowledge

A 10-item questionnaire on breast cancer knowledge developed by Loiselle and Garland for the purpose of this study was completed pre- and post- e-platform exposure. Items are based on breast cancer risk information available in the literature and PERSPECTIVE.

#### Genetic testing interest

A 6-item questionnaire, developed by Loiselle and Garland for the purpose of this study, pertains to motivation and importance of knowing one’s breast cancer risk and interest in subsequent genetic testing by asking “*would you have genetic testing?”*

#### Acceptability

The Acceptability scale developed by Tariman et al. [27] (targeting computerized health-related programs in oncology) was completed post-e-platform exposure. The scale has 6 items rated from 1 *(very difficult to understand)* to 5 *(very easy*) (α = 0.76) [27]. A score of 24 or above (80%) is considered the minimum threshold, indicating that the platform is acceptable [27].

#### Usability scale

The System Usability Scale (SUS) developed by Brooke [28] measures perceptions of e-platform usability. This self-report scale contains 10 items, each rated on a 5-point Likert scale from 1 *(strongly disagree)* to 5 *(strongly agree).* The SUS contains 2 factors: usable (α = 0.91) and learnable (α = 0.70) [29]. Converted scores can range from 0 to 100 with scores of 68 considered average [30].

#### Mood

The Positive and Negative Affect Schedule (PANAS), a self-report mood questionnaire with two 10-item scales [31] was administered pre- and post- e-platform exposure. Each item is rated on a 5-point Likert-type scale of 1 *(not at all)* to 5 *(very much)*. Separate scores are calculated for positive and negative affect and each ranges from 10 to 50. A higher total score on each scale reveals either higher positive or negative mood. Internal consistency coefficients range between 0.86–0.90 for positive and 0.84–0.87 for negative affect. Test-retest reliability (1 week) was reported to be 0.79 for positive and 0.81 for negative affect [31].

## Results

### Data analysis

Paired sample t-tests were performed to compare participant changes pre- and post- e-platform exposure for breast cancer risk knowledge, mood, and interest in subsequent genetic testing. Repeated measures ANCOVA was performed to control for potential pre-post differences in knowledge according to age and education.

### Sociodemographic characteristics

The mean age of participants was 42.6 (SD 9.5). 54.5% (*n* = 85) identified French as their primary language, 34% (*n* = 53) identified English, and 11.5% (*n* = 18) other. 64.1% (*n* = 100) reported being Caucasian and 62.8% (*n* = 98) born in Canada. 50.7% (*n* = 79) had a bachelor’s degree or higher (Table 1).

**Table 1:** Sociodemographic characteristics of participants (*N* = 156)

Characteristic	All participants (***N*** = 156)
**Mean age in years, mean (SD)**	42.6 (9.5)
**Language, n (%)**	
French	85 (54.5)
English	53 (34.0)
Other	18 (11.5)
**Country born, n (%)**	
Canada	98 (62.8)
Other	55 (35.3)
Missing	3 (1.9)
**Group self-identification, n (%)**	
Arab/West Asian	15 (9.6)
Black	11 (7.1)
Latin American	6 (3.8)
Korean	1 (0.6)
Chinese	5 (3.2)
South Asian	1 (0.6)
South East Asian	4 (2.6)
White (Caucasian)	100 (64.1)
Other	5 (3.2)
More than 1	5 (3.2)
Missing or did not report	3 (1.9)
**Highest Education, n (%)**	
High School	16 (10.3)
Technical/Vocational	53 (34.0)
Professional Degree	7 (4.5)
University: Undergraduate	50 (32.1)
University: Graduate or Post-Doc	29 (18.6)
Missing	1 (0.6)
**Marital Status, n (%)**	
Married/Common Law	106 (67.9)
Single	30 (19.2)
Widowed	2 (1.3)
Separated/Divorced	17 (10.9)
Missing	1 (0.6)

### Usage

The average PERSPECTIVE viewing time was 18.67 min (SD 0.65). For both the French and English versions, the most frequently visited pages were risk factors and risk assessment content.

### Breast cancer risk knowledge

Statistically significant pre-post changes in percentages of correct responses for breast cancer risk knowledge were obtained for obesity, genetic mutations, alcohol, risk factor stratification, breast density, and menstruation (Table 2). When age and education were controlled for, increases in knowledge for items 3 (genetic mutations) and 5 (risk factor stratification) were no longer significant.

**Table 2:** Percentages of correct responses to bca knowledge test, pre- and post-exposure to the PERSPECTIVE e-platform (*N* = 156)

Item	Breast Cancer Risk Knowledge Test (***n*** = 156)	Correct response	% Correct Pre	% Correct Post	Difference in correct responses	Paired sample t-test	Repeated measures ANCOVA
1	All women over the age of 50 have the same bca risk.	F	85%	86%	1%		
2	Obesity increases the risk of developing bca after menopause.	T	63%	99%	36%	***	***
3	Genetic mutations in genes *BRCA1* and *BRCA2* are common in women.	F	36%	62%	25%	***	
4	Drinking alcohol increases the risk of developing bca.	T	51%	99%	48%	***	***
5	In general, one risk factor has little influence on overall bca risk.	T	39%	65%	26%	***	
6	Regular physical exercise increases the risk of developing bca.	F	99%	96%	-3%		
7	A mammogram is recommended every 2 years for women between 50 to 69 years old.	T	92%	94%	2%		
8	A woman can figure out her breast density using a breast self-exam.	F	53%	77%	24%	***	**
9	Age at first period can affect bca risk.	T	39%	85%	46%	***	***
10	Doctors can estimate a woman’s bca risk level.	T	71%	90%	19%	***	***

*p < 05, **p < .01, ***p < .001

### Mood

Statistically significant changes were found in feeling interested, distressed, scared, enthusiastic (neg), proud (neg), nervous, determined (neg), and afraid after viewing the e-platform (Table 3).

**Table 3:** Mean PANAS mood scores pre- and post-exposure to the PERSPECTIVE e-platform (*N* = 156)

	Mean score pre	Mean score post	Difference in mean scores	
1. Right now I am feeling interested	3.63	4.13	0.50	***
2. Right now I am feeling distressed	1.48	1.76	0.28	***
3. Right now I am feeling excited	2.27	2.21	− 0.06	
4. Right now I am feeling upset	1.23	1.22	0.00	
5. Right now I am feeling strong	3.14	3.16	0.02	
6. Right now I am feeling guilty	1.26	1.24	−0.02	
7. Right now I am feeling scared	1.23	1.70	0.47	***
8. Right now I am feeling hostile	1.17	1.15	−0.02	
9. Right now I am feeling enthusiastic	3.16	2.72	−0.44	***
10. Right now I am feeling proud	3.08	2.76	−0.31	***
11. Right now I am feeling irritable	1.44	1.26	−0.18	
12. Right now I am feeling alert	3.23	3.22	−0.01	
13. Right now I am feeling ashamed	1.17	1.23	0.06	
14. Right now I am feeling inspired	2.91	2.94	0.03	
15. Right now I am feeling nervous	1.51	1.82	0.30	***
16. Right now I am feeling determined	3.44	3.17	−0.28	***
17. Right now I am feeling attentive	3.64	3.68	0.04	
18. Right now I am feeling jittery	1.47	1.38	−0.10	
19. Right now I am feeling active	3.01	2.86	−0.15	
20. Right now I am feeling afraid	1.29	1.75	0.46	***

***p < .001

### Understanding and interest in genetic testing

Statistically significant pre-post changes were obtained in awareness of mutations in breast cancer predisposition genes *BRCA1* and *BRCA2*, breast density, and lifestyle choices affecting breast cancer risk (Table 4). There were no significant changes in responses to how important it is to know one's breast cancer risk nor to the question “*would you have genetic testing?”*

**Table 4:** Percentages of positive responses to genetic testing questions pre- and post- exposure to the PERSPECTIVE e-platform (*N* = 156)

Item	Genetics (***n*** = 156)	Pre	Post	Difference in response	
1	I have heard about *BRCA1* and *BRCA2 (0 = No, 1 = Yes)*	28%	86%	58%	***
2	*BRCA1* and *BRCA2* are: *(0 = Incorrect, 1 = Correct)* *a) Medical equipment used to perform breast cancer screenings* *b) Breast cancer stages* *c) Hormones* *d) Genes* *e) Breast tissue measures*	49%	80%	31%	***
3	How important to know level of bca risk? *(1 = Not at all, 7 = Extremely)*	6.30	6.25	−0.05	
4	Women with denser breasts: *(0 = Incorrect, 1 = Correct)* *a) Have a higher risk of developing breast cancer* *b) Have a lower risk of developing breast cancer* *c) Breast density does not matter*	25%	74%	49%	***
5	Would you have genetic testing? *(1 = Very Unlikely, 7 = Very Likely)*	5.18	5.47	0.29	
6	Do you think your lifestyle choices can change your bca risk? *(0 = No, 1 = Yes)*	84%	98%	14%	***

*** *p* < .001

### Acceptability

The total mean score for acceptability was 26.19 out of 30 (SD 0.157). ‘How helpful was this e-platform in providing information on breast cancer risk" and overall satisfaction were rated highest, 4.60 and 4.43 respectively, on 5.0 (Table 5).

**Table 5:** Mean Acceptability e-Scale ratings of PERSPECTIVE e-platform (*N* = 156)

Item		1 (low) – 5 (high)	SD
1	Easy to use	4.40	.825
2	Understandable	4.35	.785
3	Enjoyable	4.15	.917
4	Helpful	4.60	.725
5	Time spent was acceptable	4.25	.742
6	Overall satisfaction	4.43	.591
	**Total**	**26.19**	**.153**

### Usability

The mean total score for the SUS was 42.85 out of 50 (SD 0.267). ‘Did not have to learn new technical skills’, and ‘Information was easy to understand’ were the SUS items with the highest scores, 4.62 and 4.46 respectively on 5.0 (Table 6).

**Table 6:** Mean System Usability Scale ratings of PERSPECTIVE e-platform (*N* = 156)

Item		1(low) - 5(high)	SD
1	I would like to use this website frequently	3.60	.956
2	I found the information on the website easy to understand	4.46	.748
3	I thought the website was easy to use	4.29	.852
4	I think I could use the website without technical support	4.38	.798
5	I found that the website features were well put together	4.24	.788
6	The content presented on the website showed no contradictions	4.26	.763
7	I think that most people would learn to use this website quickly	4.34	.732
8	Going through the website did not require effort	4.28	.893
9	I felt confident in using the website	4.38	.774
10	I did not have to learn new technical skills to use this website	4.62	.749
	**Total**	**42.85**	**.267**

## Discussion

Phase 1 of PERSPECTIVE presented herein used the breast cancer risk stratification e-platform as a pre-assessment tool, among women with no actual knowledge of their personal breast cancer risk. Within this context, we explored its usefulness as a potential decision aid tool, acknowledging women's important role in decision-making. More specifically, this study sought to document women’s perceptions of PERSPECTIVE through assessing potential changes in breast cancer risk knowledge, mood, understanding and interest in genetic testing, as well as acceptability and usability ratings of the e-platform.

Results indicate that following e-platform exposure, breast cancer risk and genetic testing were better understood by participants. Significant increases in their knowledge of breast cancer risk, specifically obesity, alcohol, breast density, menstruation, and the risk estimation process remained even when age and education were controlled. These findings are consistent with previous studies revealing that interactive health tools can inform the public of disease risk, as well as increase the processing and uptake of relevant breast cancer prevention information [32–34]. The literature also supports the added benefits of validated breast cancer screening decision aids [35–37]. It is important to acknowledge, however, that the primary focus of this study was to begin to document how participants interacted with the e-platform, engaged in information gathering and perceived the potential benefits of the e-platform. Actual decision-making processes will be addressed in a follow-up study.

Participants’ answers to “*would you have genetic testing?”* did not change pre-post e-platform exposure. We acknowledge that decisional processes related to genetic testing are complex and affected by several factors including context, age, income, marital status, health status, family history, locus of control, and anxiety [38]. With regards to sociodemographic characteristics considered in this study, younger age (under 50) and marital status (married or common law) were linked to significantly lower interest in testing. However, data on breast cancer family history and locus of control previously found to significantly affect decisions about genetic testing, were not collected [38]. Lack of interest in genetic testing may also be related to mood; post assessment, participants reported a range of mixed emotions including interest, distress, and being scared demonstrating concerns related to breast cancer – which may influence the importance placed on the information provided and subsequent decision-making. Changes in mood highlight the importance for healthcare professionals to provide context for gauging the appropriateness of genetic counseling services. As such, a high quality e-platform should be used as a complement to more formal genetic counseling rather than as a substitute.

Usability and acceptability scores revealed that the e-platform met the informational needs of women in the general population for whom it was intended. Taken together, these findings support the relevance of quality e-tools to providing evidence-based and stratified breast cancer screening information [39–41].

### Limitations

This study has several limitations including a short pre-post measure timeline (i.e., 30 min). Whether changes in knowledge were sustained and interest in genetic testing evolved overtime cannot be ascertained. With any convenience sampling, women who chose to take part in this study were presumably interested in the topic and this may have biased findings. In addition, whereas the e-platform was designed for various literacy and educational levels, participants’ overall educational levels were higher than the general public [42].

## Conclusions

An informative e-platform directed specifically at women in the general population can increase awareness, knowledge and support informed decision making for women in the breast cancer risk stratification process. The study provides insight into important features of e-platforms that provide health and illness-related information, as well as relationships between affect and  breast screening information. In accordance with the shift towards personalization of health care according to patients’ needs and preferences, tailored recommendations based on personal breast cancer risk present an important contribution to the field. The e-platform developed and evaluated herein represents a first phase. Phase 2, which involves the integration and implementation of PERSPECTIVE is currently underway. A version of the e-platform will be assessed among  women undergoing genetic testing to determine their polygenic risk score from low penetrance common genetic variants. The e-platform will incorporate screening and follow-up recommendations into its features – being both practical and convenient.

## Data Availability

The datasets generated during and/or analysed during the current study are not publicly available at this time but are available from the corresponding author on reasonable request.
